# Amyloid Biomarkers in Conformational Diseases at Face Value: A Systematic Review

**DOI:** 10.3390/molecules23010079

**Published:** 2017-12-29

**Authors:** Maria Fernanda Avila-Vazquez, Nelly F. Altamirano-Bustamante, Myriam M. Altamirano-Bustamante

**Affiliations:** 1 Unidad de Investigación en Enfermedades Metabólicas, Centro Médico Nacional Siglo XXI, IMSS, Mexico City 06720, Mexico; feravilaibero@gmail.com; 2 Health Department, Universidad Iberoamericana, Mexico City 01219, Mexico; 3 Instituto Nacional de Pediatría, Mexico City 04530, Mexico; nellyab34@gmail.com

**Keywords:** biomarker, conformational diseases, Alzheimer’s disease, diabetes mellitus, amyloid oligomers, cytotoxic oligomers

## Abstract

Conformational diseases represent a new aspect of proteomic medicine where diagnostic and therapeutic paradigms are evolving. In this context, the early biomarkers for target cell failure (neurons, β-cells, etc.) represent a challenge to translational medicine and play a multidimensional role as biomarkers and potential therapeutic targets. This systematic review, which follows the PICO and Prisma methods, analyses this new-fangled multidimensionality, its strengths and limitations, and presents the future possibilities it opens up. The nuclear diagnosis methods are immunoassays: ELISA, immunodot, western blot, etc., while the therapeutic approach is focused on pharmaco- and molecular chaperones.

## 1. Introduction

How does evolution make proteins fold so fast? This is an unresolved problem at the crossroads between chemistry, physics, biology and proteomic medicine [1,2]. Sometimes proteins do not fold correctly and present folding errors (anomalous folding of non-native structures (NN)) [3,4,5]. Non-native structures interact with each other and form intra- or extracellular aggregates that may eventually form amyloid oligomers, protofilaments, amyloid fibers, leading to a resulting group of health problems [6,7,8,9,10,11]. Some examples are neurodegenerative diseases like Alzheimer’s disease (AD) [12,13,14,15], and Parkinson’s disease [16]. Another group are chronic diseases like cancer, cardiovascular diseases, and diabetes mellitus type 2 (DM2) [12,17]. Carrell grouped these diseases and called them conformational diseases (CDs) [18,19]. CDs have a common pathophysiological basis—an alteration at protein level, whether in size, shape, folding or conformation. Consequently, the resulting cytotoxicity produces a deficiency of functional proteins [1,5,6,7,11,19,20].

The main component of amyloid deposits in CDs, such as AD, are oligomeric forms of amyloid-β (Aβ) such as Aβ1-40 and Aβ1-42, whereas in DM2 the deposits are from the amyloid polypeptide of the islets, also known as amylin [12,15,21]. Amylin is a hormone excreted and expressed synchronously with insulin in β-cells of the pancreas [8]. These amyloid forms have conformational polymorphism (dependant of the microenvironment) which produces different degrees of cytotoxicity [11,22].

The β-cross sheet is the structure that is formed by exposing the hydrophobic regions of soluble proteins giving rise to non-native structures. The NN structures interact with each other to form soluble oligomers that remain stable towards aggregation and oligomerization. This explains the entrenchment and deposition of protein aggregates in various organs, whereby tissue damage and organic dysfunction occurs [5,6,11,23].

Numerous studies show evidence of the interrelationship between CDs and obesity as a risk factor for DM2 and this, in turn, for AD [17,24]. In general, studies suggest that increased adipose tissue affects the body’s response to insulin, leading to insulin resistance, impaired glucose metabolism, and faulty lipid homeostasis. This contributes greatly to heart disease since patients also develop hyperinsulinemia and hyperamylinemia which, in turn, leads to proteotoxicity and β-amyloid protein deposition [25]. A study by Zhao et al. with human pancreas samples proved the presence of oligomers in the pancreas through immunofluorescent staining [26]. AD, for example, affects approximately half of the population over 85 years of age [27]. Other theories state that such diseases may be due to a mutation [28], increased stress in the amyloid precursor protein (APP)/Aβ metallic transport system, lifestyle [29], and promotion of ROS [30]. Another important factor is poor processing of the APP [31] influenced by β-proteins (BACE1) and γ-secretases [32], which promote protein aggregation. This information reveals the importance of amyloid deposition’s importance in global health [1,2,23].

For this reason, it is important to develop diagnostic techniques that can detect conformational diseases before their onset. It is believed that early diagnostics can be achieved through biomarkers even before CDs symptoms appear. A biomarker is an indicator of normal biological processes, pathological processes or pharmacological responses to a therapeutic intervention. In AD the APP and Aβ-protein are the biomarkers that have tested the ‘amyloid hypothesis’ and have led to therapeutic interventions [33,34]. Biomarkers used for diagnosis are not only found in brain tissue, but also in peripheral tissues and fluids (i.e., cerebrospinal fluid (CSF)). However, these have limitations: oligomer stability [24], sample amount [27], assay number [35], sensitivity differences, and specificity differences. In addition, most of the diagnostic techniques require that the disease be at an advanced stage and often the diagnosis does not occur until the post-mortem stage with the presence of amyloid deposits in brain tissue [36].

**Figure 1:**
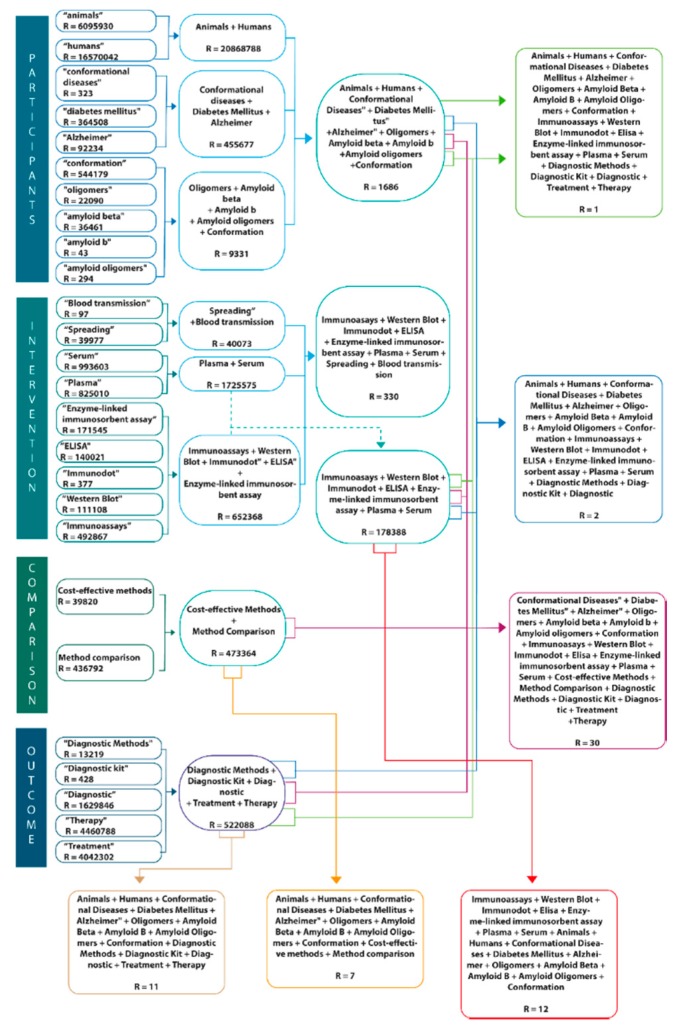
PICO approach for systematic review. P (participants): Humans, animals, Alzheimer, Diabetes Mellitus, conformational diseases, amyloid oligomers, amyloid-β, oligomers, conformation. I (intervention): immunoassays, western blot, immunodot, ELISA, Enzyme-linked immunosorbent assay, plasma, serum, spreading, blood transmission. C (comparison): Method comparison and cost-effective O (outcome): Diagnostic methods, diagnostic kit, diagnostic, treatment, therapy.

**Figure 2:**
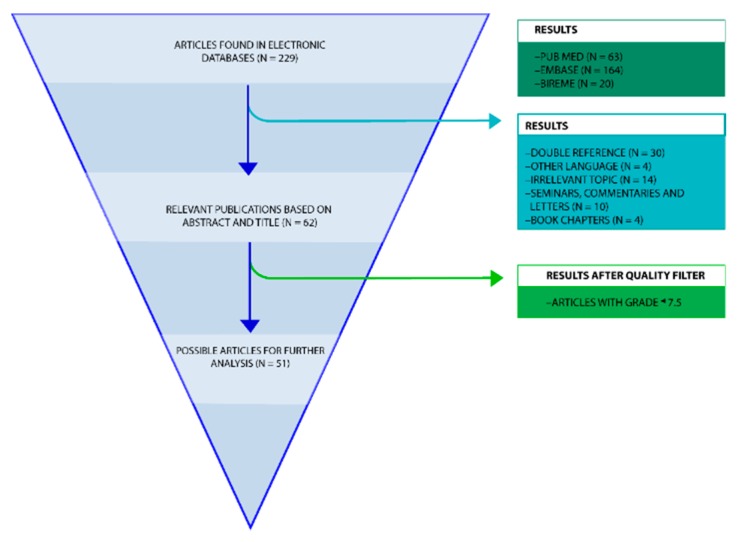
Flowchart review process. A PRISMA flowchart of the systematic review on the diagnostic methods for conformational diseases related to amyloid oligomers and the multi-target proteins using as potential drugs for CDs.

The purpose of this review is to evaluate—following the Participants/Intervention/Comparison/Outcome (PICO) and Preferred Reporting Items for Systematic Reviews and Meta-Analyses (PRISMA) methods—the literature related to immunoassay methods used to measure different amyloid biomarkers, with an emphasis in enzyme-linked immunosorbent assay (ELISA), immunodot, and western blot (Figure 1 and Figure 2). One of the main objectives of this review is also to discuss new information in the latest findings about the aggregation of β-amyloid oligomers as well as the techniques for their diagnosis. Moreover, this review intends to include a broad spectrum of the status of amyloid hypothesis, diagnostic and treatment techniques. Given that Alzheimer’s disease and diabetes mellitus are the costliest, both economically and socially, they are the most relevant diseases for this review. The following research questions based on the PICO method will be addressed: how are the different diagnostic methods for these conformational diseases related to β-amyloid oligomers and to what extent does their use as a multi-target technique provide a reasonable foundation for treating CDs?

## 2. Results

The review process began with 229 references retrieved from a variety of electronic databases (Figure 2). Of these 229 articles, 63 were retrieved from PubMed, 164 from Embase, and 20 from Bireme. However, not all of these articles were relevant to the research topic. For this reason, 192 articles were discarded. After that, articles with a double reference (*n* = 30); in another language (*n* = 4); whose topic was irrelevant to the review (*n* = 14); seminars, commentaries or letters (*n* = 10); and book chapters (*n* = 4) were discarded. This selection process left 51 articles of use to the review (Figure 2).

A second sifting was performed whereby only the articles that obtained over 75 points in the quality criteria were considered, the criteria used is similar to the one described by Van Mol [37] where the articles are ranked in relation to specific quality standards such as having a specified aim, a detailed description of methods, valid and reliable measuring, etc. Table 1 shows the result of each of the 51 articles that were considered. Following this process, 51 articles on immunoassays and β-amyloid oligomers were read in full text and further analysed (Figure 2), Table 1 list all 51 articles and their characteristics.

In general, the articles discussed the use of β-amyloid as a biomarker. In order to come to this conclusion, many studies performed a series of immunoassays, the most commonly used of which were ELISA and the western blot ones. However, a number of studies used immunodot, dot blot, homogeneous time-resolved fluorescence immunoassay (HTRF), and mass spectrometry (MS).

Most articles gave a detailed account of the type of immunoassay used, incubation time, number of washes, block solution used, pre-treatment or dilution, and the molecule analysed, all of which was relevant to the aim of this review which is to get to know the different methods of analysis. The most commonly analysed molecules throughout the articles were β-amyloid, APP, microtubule-associated protein tau (T-tau), amylin, HEWL oligomers, and antibodies.

Of the 51 articles taken into consideration at the third stage, 17 of them did not provide information on β-amyloid oligomers in human tissues and were actually reviews about specific topics: molecular differences between oligomers, the role of metal ions in β-amyloid aggregation and toxicity, and various treatments. It is also important to mention that articles found in this review are from different countries of origin; furthermore, it is unclear which country did more research on β-amyloid diagnostic methods, however, it can be stated that a slightly higher percentage of the articles came from Asian countries. The target populations were similar across the articles, most sampled humans with Alzheimer’s disease CSF, human plasma, synthetic β-amyloid proteins, serum antibodies, and cerebral tissue with β-amyloid deposits. Interestingly, the other most commonly used samples were those of mice and rats, for instance, one of the samples mentioned were the brain samples of passively immunized 3× Tg-AD mice control and wild-type [38,39,40].

### 2.1. The Biomarker Dilemma

**Figure 3:**
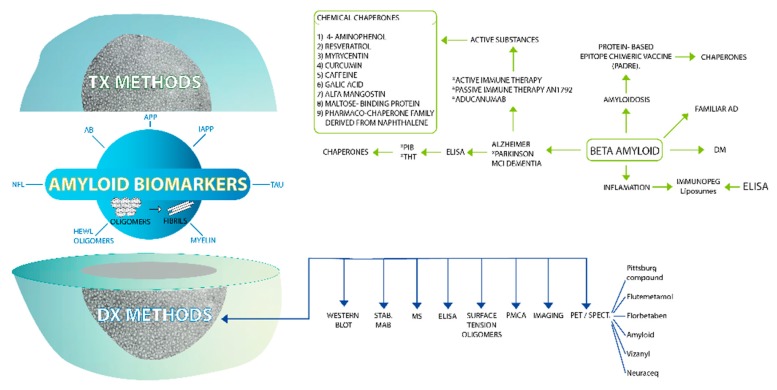
Amyloid biomarkers as target molecules for diagnostic methods and therapeutic approaches in conformational diseases. If we open the amyloid biomarker nucleus we will find several polymorphic amyloid molecules of different target proteins such as APP, AB, amylin, etc. These polymorphic amyloid molecules lead developing actions for diagnostic methods such immunoassays (ELISA, WB, immunodot, etc.), PET/SPECT with several reagents. In relation of multi-target therapeutic drugs, the main explored therapeutic approach is the use of chemical chaperones.

**Figure 4:**
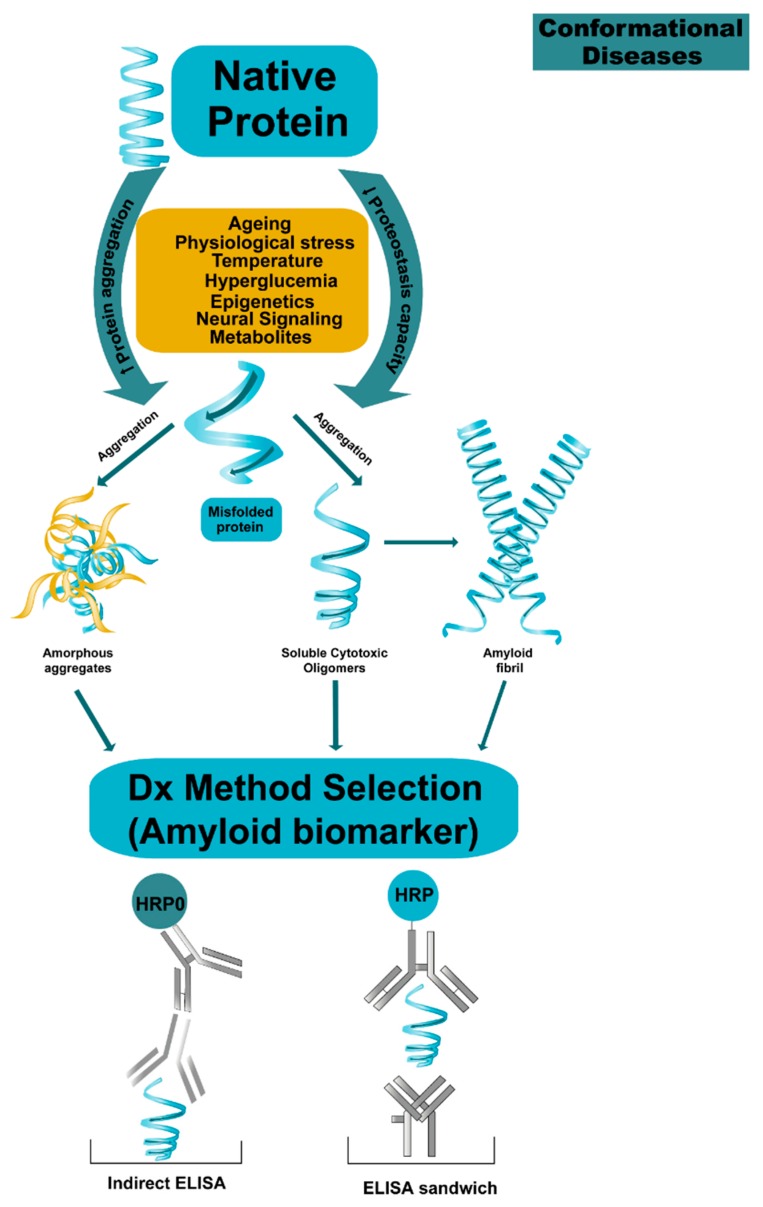
Proposal mechanism on protein aggregation in conformational diseases. The proteins lose their native state by multidimentional factors such as ageing, epigenetic, etc., which produce proteostasis collapsed by increasing protein aggregation and decreasing the capacity for proteostasis. Monomers can self-aggregate and form cytotoxic oligomers leading to self-aggregation or hetero-association and finally the formation of fibrils. The process is dynamic, the oligomeric species become a multi-target molecules for early diagnostic and treatment and the challenge is to claim and stabilize them.

**Figure 5:**
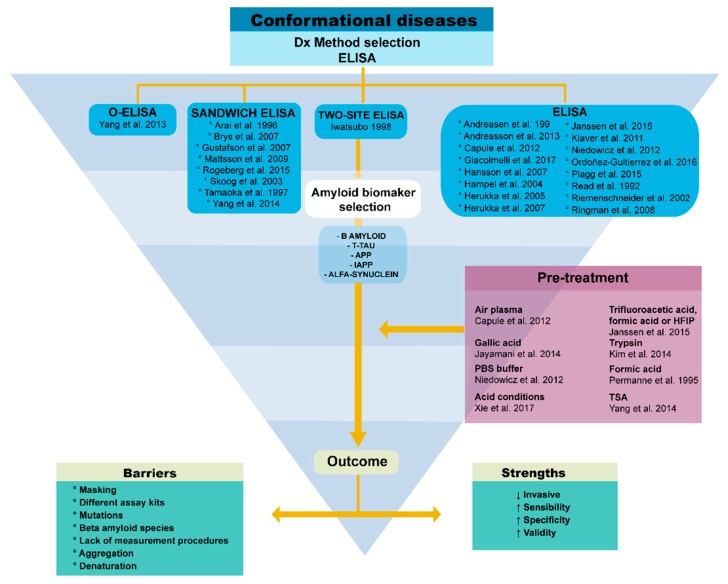
The ELISA challenges and evolving solutions. We show the strengths and limitations of this immunoassay and the pre-treatment possibilities to overcome the limitations.

According to the amyloid hypothesis the stages of β-amyloid aggregation disrupt cell-to-cell communication and activate immune cells. These immune cells trigger inflammation, producing a continuous state of inflammation which ultimately leads to the destruction of the brain cells [13]. Because of this, the biomarkers proposed to do an early diagnosis of AD include central nervous system (CNS) Aβ42, Aβ42/Aβ40 ratio, soluble APP isoforms (sAPPα and sAPPβ), T-tau, phosphorylated tau protein (P-tau), total tau (T-tau) protein, and neurofilament light (NFL) protein (Table 1 and Table 2, Figure 3, Figure 4 and Figure 5).

In 1992 Read et al. studied one of the first biomarkers: APP. It competes with the beta Tau protein, also an early biomarker for effective AD diagnosis [42]. It is important to mention that the most common method used is ELISA, and, as can be seen from the studies, a statistical analysis of correlation is required in order to evaluate the validity of biomarkers.

However, these biomarkers are usually not entirely helpful in the diagnosis of AD. This means that the biomarker is not the only element affecting the results, as other factors such as the tissue samples used for amyloid oligomer measurement affect the detection of these molecules (i.e., blood, cerebrospinal fluid, and brain tissue) [40]. However, it has been proven that, in patients with AD, Aβ and β-tau protein levels are higher than APP or Aβ42 in CSF, which is why these levels are used as a biomarker of AD [34]. Other studies support this conclusion: a longitudinal multicenter study with 196 mild congnitive impairment (MCI) patients, 37 of whom developed AD had these Aβ42 and T-tau levels. This study had a sensitivity of 89% for MCI casas with progression to AD [40] (Table 2, Figure 3, Figure 4 and Figure 5).

Similarly, a longitudinal MCI-control study in patients with AD proved that low CSF AB42 and high CSF T-tau is a characteristic of AD patients. What’s interesting about this study is that the sensitivity was almost 88% and had a specificity of 80%, making the CSF-B-amyloid levels measured with ELISA a very sensitive diagnostic marker [39]. In the Zetterberg et al. study, a sensitivity of 68% and a specificity of 97% was reported, using sample biomarkers. The positive predictive value in other studies reached 94% and a negative predictive value of 81% [86]. Other studies that support this diagnostic method have used ELISA to detect β-amyloid protein in CSF and T-tau [51,52,53,54].

The most important finding is that this protein can illustrate the pathological changes of AD using only this molecule as a biomarker [69]. Even though there are a great variety of biomarkers, there are still novel ideas on where these amyloid oligomers can be measured. One example is platelets in mutated mice [68]. Another example of biomarker used is the T-tau/Aβ42 ratio. In a longitudinal control study 43 controls were assessed with Luminex reagents and four of these developed MCI. Abnormal levels of this ratio were found in adults over 53 years old. Moreover, controls had increased frequency of the e4 allele of the apolipoprotein E gene and increased risk of progression to MCI. This study was useful in order to give patients a risk-benefit analysis, in order to prevent further onset of MCI and dementia [62] (Table 2, Figure 3 and Figure 4).

A 2-year longitudinal study examined five commonly used CSF biomarkers (isoprostane (IP), T-tau, P-tau, Aβ42, and Aβ40) for AD in a study of healthy controls, stable MCI and MCI patients who later had AD. In all initial measurements and follow-up measurements of these biomarkers most of the patients with stable MCI and MCI had an evident decline of CSF biomarkers. P-tau and T-tau were the strongest biomarkers and those with less variability. This information adds to the biomarker dilemma. Moreover, this could also make scientists question which are the most convenient biomarkers to use in control and AD patients [45].

This dilemma has some other implications. In some research, the measurement of β-amyloid proteins 42 and 40 are one of the most reliable biomarkers for AD, but not for serum amyloid protein samples, according to Iwatsubo et al. [28] and Klaver et al. [60] Some of the barriers include the “masking” of the oligomers when, while binding to an antibody, they lose signal. Moreover, most of the time there are not enough samples in order to perform all the necessary tests. More importantly, laboratories use different methods for ELISA immunoassay, and sometimes different assay kits (Figure 3 and Figure 4). Part of the biomarker use comes from the βAPP have a mutation located in the amino (N)- and C-flanking regions of the Aβ domain and increase the secretion of total Aβ or the ratio of Aβ42. These results prompted researchers to examine if the levels of Aβ, especially those of Aβ42, which are altered in the plasma of patients with AD, including carriers of APP717 (Val to Ile) mutation that is linked to familial AD. A similar study with genetic mutations performed by Ringman et al., was used to study biomarkers and how these can be applied to the diagnosis of presymptomatic diseases. Some of the conclusions were that Aβ42 is elevated in plasma, specifically in familial Alzheimer’s disease (FAD) mutation carriers, and this level can decrease with disease progression. The ratio of Aβ42 to Aβ40 was reduced in the CSF of non-demented MCs and the elevation of T-tau and P-tau are sensitive indicators of presymptomatic disease [70] (Figure 3, Figure 4 and Figure 5, Table 2).

These results can be used for the biochemical diagnosis or classification of subtypes of AD. One of the limitations for amyloid oligomer measurement is the presence of a mutation, therefore, methods are needed in order to identify or discriminate between different types of β-amyloid species [28]. Another important factor is the considerable overlap of CSF-Aβ42 values when compared between patients with AD and controls. Sometimes the disease’s stage and amyloid oligomer species can affect the detecting ranges and the values themselves [81]. Some ELISA assays have detected different forms of APP including secreted APP forms [68]. Sometimes these results can cause confusion in the results’ reading (Figure 3 and Figure 4, Table 2). In our group we have demonstrated that the rat IAPP hexamers are diagnostic biomarkers of the onset and progression of diabetes mellitus and play a role as therapeutic targets [20,22].

### 2.2. Technique Challenges and Their Evolving Solutions

Even though the study of β-amyloid seems complicated, scientists have developed different techniques to measure and use them as diagnostic information. For example, the most common techniques are immunoassays, imaging, and histological methods. Immunoassays are methods of special interest since they are low cost and less invasive. ELISA can detect protein concentrations due to a series of exposures to antibodies without the use of radioactivity or fluorescent properties. However, this method has a lot of variabilities and some limitations. One limitation is the lack of a reference measurement procedure (RMP) [35]. Another limitation is “antibody masking” which prevents complete antibody measurement by ELISA [60]. Nevertheless, new ELISA techniques are being developed to make the process more efficient and capable of detecting different types of molecules [36]. For instance, competitive ELISA prevents the denaturation of β-amyloid protein—a very common problem (Figure 3, Figure 4 and Figure 5).

Another example is the formulation of a pre-treatment protocol to prevent aggregation of amyloid β-protein. The result is a greater amount of measurable Aβ [56]. Also, techniques like, “two-site” ELISA, are a new trend. In this technique the same antibody is used with a specific sequence for uptake and detection [65]. Another new technique is the use of antibodies using STAB-MAb bound to PEGylated liposomes [32]. Some new protocols for o-ELISA have been developed for soluble oligomers for β-amyloid protein in the human brain. This is proof that the use of immunoassays is a great tool to identify biomarkers for AD and at the same time identify the distinct species of β-amyloid proteins. This method is interesting because of the use of different antibodies [84] (Figure 4 and Figure 5).

Another technique used as an alternative to other methods that require sample handling, several washes and showed increasing inaccuracies and long working times is HTRF [47]. One of the new methods for diagnosis is the use of Positron Emission Tomography (PET) and single-photon emission computed tomography (SPECT). These methods use biomarkers based on fluorescent molecules such as thioflavin T (ThT), the Pittsburgh compound B (PiB) [66], flutemetamol and fluorbetaben [48]. However, only three have been approved by the FDA in the United States: Amyvid™, Vizamyl™ and Neuraceq™ [33]. The problem with these methods is their specificity, or lack thereof since fibres, myelin and neurofibrillary tangles (NFT) are marked along with the molecules relevant to diagnosis. For this reason, it is important to perform analyses for different types of amyloid deposits [67]. Within this type of measuring method, selected reaction monitoring mass spectrometry (MS) is also used [59]. Sometimes the sample collecting in CSF are invasive and costly, for this reason researchers have chosen to find reliable and low-cost biomarkers in plasma, monocytes, and platelets [68] (Table 2, Figure 3, Figure 4 and Figure 5).

Techniques such as mass spectrometry have also been used in conjunction with immunoassays. However, they don’t present great advantages [59,71]. Methods for determination of Aβ protein deposition are Bodian or Bielschowsky. Both of these, along with neuropathological studies, have a high correlation when compared with immunoassays. This proves that immunoassays are an effective diagnostic technique [67]. The measurement of surface tension oligomers is also a new trend; it identifies the hydrophobic properties of the amyloid and aggregate rHL fibers. This technique is non-invasive and works well with highly diluted solutions. It is believed that changes in proteins are due to variation in insulin hydrophobicity [73]. One of the limitations is that the oligomers of the amyloid β-protein have the same structure as the native Aβ protein. For this reason, studies aim to obtain the poorly folded form. For this reason, techniques like protein misfolding cyclic amplification (PMCA) have been developed [74] (Table 2, Figure 3, Figure 4 and Figure 5).

Despite the different studies, there are still doubts about the reliability of β-amyloid in CSF and serum as a biomarker [81]. Researches have studied the relationship between the Aβ protein and oligomer levels and different stages of Alzheimer’s disease (AD). Within these investigations there is also the study of a possible AD treatment. For example, peripheral administration of anti-6A 15-T serum antibodies mitigated AD-like pathology and cognitive decline of aged 3× Tg-AD mice with passive immunization. This intervention also effectively reduced the levels of the most toxic form of amyloid for neurons: soluble oligomers. Passive immunization significantly reduced the insoluble amyloid and plaque load in the brains of 3× Tg-AD mice. Antibodies generated or administered in AD model animals or clinical trials have been shown to reduce amyloid deposits in the brains. This could be measured by western blot, immunodot and ELISA [38]. Another possible treatment is trechostatin, also explored with western blotting and binding to β-amyloid protein [85].

This information provides a perspective in how important the diagnosis of β-amyloid deposits in human tissues is. Moreover, finding a treatment and low-cost intervention for different patients with AD would also be beneficial. However, the different analyses used to measure the protein interest are still being revised and standardized. For this revision, only the immunoassay ELISA was analysed, since it is one of most cost-effective methods (Table 2, Figure 4 and Figure 5).

As already mentioned, the different uses of ELISA techniques can affect the biomarker values for measurement. In the past, most of the diagnostic techniques used clinical methods, having approximately an 88–90% accuracy. ELISA immunoassays are trying to compete with these values for diagnosis. Due to this, there are some pre-treatment implications and methodological components that need to be taken into account, for instance, the uniformity of lab and measurement results is a barrier despite the fact that in some of the latest results there is a constant specificity and sensitivity of the ELISA assays from almost 90%. These results are very optimistic; however, these need to ensure longitudinal consistency. Similar studies report having a big variability between and within laboratories 10–15% [40] (Figure 4 and Figure 5).

Another consideration is that ELISAs are designed for monomer detection [56,84], but sometimes these monomers tend to aggregate and affect the measurement ranges of the ELISA. In addition, the sensitivity to detect natural oligomers is not well established. Alternative methods like western blot, usually need denaturation to see the peptides and this could decompose oligomers into monomers [84] which this is not a desired practice (Figure 4 and Figure 5).

Antibodies are used in ELISA in order to identify the protein of interest. The antibodies used are a potential problem. When the antibodies are added to the substrate, the anti-Aβ antibodies bind to serum Aβ, which can reduce ELISA detection of these antibodies. In some of the experiments, the use of anti-Aβ with different affinities and K constant has been criticized, but the results confirm that the use of these antibodies have nothing to do with the different concentration results [60]. The use of different ELISA kits is a big factor for biomarker measurement results [40] because the measuring range can vary. One example is the use of Human Amyloid β (1-x), which has specific characteristics and different forms of application to an ELISA protocol. Another example is the IBL International Assay Kit, which has a detection range between 7.81 and 500 pg/mL and can detect Aβ forms of various lengths, ranging from 28 to 42 amino acids, provided they show no *N*-terminal modification. The cross reactivity with *N*-terminally reported modified Aβ amounts to 0.1% [56,68] (Table 2, Figure 4 and Figure 5).

New techniques try to overcome the binding-antibody problem in relation to materials used for ELISA and pre-treatment. In the same way other possible pre-treatments are the use of acid (trifluoroacetic acid (TFA), formic acid (FA), and hexafluoroisopropanol (HFIP)). In one experimental report, this was first done with synthetic Aβ monomer and then with biological brain extract of APP23 mouse model for AD and human cerebrospinal fluid of AD patients and control individuals. These three groups showed minimal deviation (<10%) from the theoretical standard concentration, demonstrating no adverse effect on the samples [36] (Table 2, Figure 3).

ELISA is the more cost-effective technique. Some of the reasons are that it doesn’t need a very large number of personnel. One person can perform the pre-treatment and the final measurement of oligomers. The most important equipment is the UV-reader, which can be installed in any laboratory. Moreover, the general ELISA protocol is very simple. Technicians do not need any personalized or specialized training for this assay, but not only the protocol elements should be revised for β-amyloid measurements. Oligomerization of Aβ can lead to epitope masking and steric hindrance, which can result in an underestimation of the Aβ concentration in ELISA analysis. Reconstitution in phosphate-buffered saline (PBS), TFA, HFIP or FA is one of the possible pre-treatment techniques that can solve this problem. Different tests have proved that the best solution for pre-treatment is 1% NH_4_OH [56] (Table 2, Figure 4 and Figure 5).

There are some protocols where the assay can selectively measure natural Aβ oligomers of various sizes. Two o-ELISA, validated its specificity for oligomeric Aβ and then showed their utility when applied to human and mouse brain tissues [84]. These ELISAs can selectively quantify synthetic and natural oligomers of human Aβ over a wide analytical range. Moreover, they selectively quantify brain Aβ oligomers from dimers up to much larger assemblies, but cannot detect monomers. The o-ELISAs revealed 1000-fold more oligomers than monomers in the AD cortex and documented the rise in Ab oligomers in APP mouse brain with age. CSF gave no o-ELISA signal, suggesting that the hydrophobicity of oligomers makes them very low or absent in aqueous fluids (Table 2, Figure 3).

### 2.3. Polymorphic Amyloid Structures Implications

Even when the protocols and the use of different biomarkers for β-amyloid oligomers have been investigated, the biochemical implications play an important role in the disease diagnosis and the performance of ELISA protocols. The secondary structure of the β-amyloid protein seems to be the one with more interest, since it is the form used for ELISA assays [36]. Moreover, the oligomerization in samples can affect the accuracy of ELISA measurements of the total β-amyloid protein [56]. This may have a similar effect to amyloid aggregation as a barrier for oligomer measurements. For this reason, the different species of the Aβ protein need different modifications in the measurement techniques [66]. This represents a difficulty, since not all laboratories have the material and equipment to discriminate between the different molecules.

The use of chemicals could have an impact on the biological samples; however, it has been observed that some of the most important factors are the drying times and reconstituting the samples, rather than the chemical itself [56]. If the times of drying are too long, it is likely that the protein of interest denaturizes and thus, the concentration results could be altered.

In the same study performed by Brys et al., a set of five of the most common biomarkers were used to identify the levels of these biomarkers in relation to the onset of AD. One of the interesting findings was that these studies were able to tell which biochemical composition of CSF reflected the AD pathology. For example, some of the characteristics observed were the neurofibrillary tangles, amyloid plaques, and oxidative damage to neuronal cell membranes [45]. In addition to this, the sensitivity and specificity of the measured biomarkers were almost 80%, which can give a clue to which biomarker is better to use in order for early AD diagnosis.

### 2.4. Multi-Target Therapeutic Approaches

ELISAs have been used in the investigation field for a long time. However, with new technologies there is more use of imaging for β-amyloid aggregation diagnosis. Some of the agents used are PiB, ThT, which need ELISAs in order to quantify the binding of these agents with the β-amyloid oligomers. These methods tend to be more expensive than immunoassays. What could really benefit patients? One example is that treatment could be possible if measurement ranges are standardized for all patients and for the different AD stages and age [40]. Moreover, the early prediction of Alzheimer’s onset would really benefit patients before the symptoms appear. A population-based longitudinal cohort study supports this idea. In said study, 35 non-demented 85-year-old adults underwent a LP and were followed for 3 years. The results show that low CSF AB42 levels were correlated with predicted progression towards dementia [77]. A similar study was conducted by Herukka et al., the difference was that some of the patients in the study had already been diagnosed with Alzheimer’s disease [55]. Moreover, abnormal values at baseline have higher risk of developing AD [44,50,80].

Scientists are testing a number of strategies to block the effects of β-amyloid. Several drugs targeting β-amyloid have reached human clinical trials. Until the successful aducanumab trial published in 2016, there was no clear indication that these drugs moderated brain changes brought about by Alzheimer’s or protected brain cells. Aducanumab, an antibody that binds to both insoluble forms of β-amyloid (amyloid plaques) and soluble forms, reduced levels of β-amyloid in the brain and slowed the rate of cognitive decline in a group of people who had mild or preclinical Alzheimer’s disease [13]. Active and passive immunotherapy may limit cerebral Aβ deposition or accelerate its clearance. Another technique used in a clinical trial were the monoclonal anti-Aβ antibody used to construct immune-PEG-liposomes in order to capture Aβ in the peripheries. The role of ELISA here was very important. ELISA was used to test affinity to STAB-Mab for Aβ peptides, to see if the immune-PEG-liposome bind to the protein of interest [32] (Figure 3).

In another clinical study, ELISA was used to measure the initial levels of Aβ protein in brain and the levels after the treatment. Recently, in the preparation of a protein-based epitope chimeric vaccine using six copies of Aβ1-15 fused with the pan HLA DR-binding peptide (PADRE), generated an adequate anti-Aβ42 antibody response and provided a protective effect in AD model mice. It also possessed the therapeutic potency of inhibiting Aβ42 oligomer-induced neurotoxicity. Later the efficacy of passive immunization with the Aβ42 oligomer conformation-sensitive serum antibodies in 3× Tg-AD mice after epitope mapping of serum antibodies was evaluated and characterized [38]. These sensitive and specific ELISAs can be used in quantitative biochemical pathology to correlate oligomeric forms of Aβ for mechanistic analyses and further fluid biomarker searches [84] (Figure 3, Figure 4 and Figure 5).

Previous attempts at therapeutic intervention in AD focused on reducing the levels of brain Aβ by using antibodies against Aβ or by passive immunization with anti-Aβ antibody. Antibodies' experiments suggested that antibodies against Aβ can modulate the transfer of Aβ between the brain and plasma. Therefore, compounds that can bind to Aβ in the periphery without penetrating blood brain barrier may be ideal candidates as effective therapeutic agents for AD. One such candidate is gelsolin of 90 kDa protein, which is present as a circulating and also an intracellular protein [85].

Recently our research group identified a meta-structure called meta-pharmacophore, which allows the development of novel drugs [22]. We described a pharmaco-chaperone family derived from naphthalene which can do a variety of things: some either accelerate or inhibit the protein-aggregation process (depending on their concentration level), some stabilize the native conformer, some stabilize fibres, and other bind to oligomers accelerating fibre formation. The pharmaco-chaperones act as modulators that provide dynamic interventions and the multi-target capacity required to meet the treatment challenges of conformational diseases [22].

## 3. Discussion

Just like the progression of AD, the progression of conformational diseases is an important topic for global health. Because amyloid β-protein deposition begins to accumulate in obese patients’ hearts and in the early stages of insulin resistance [25], it is important to seek timely treatment. Although there are still questions about whether β-amyloid protein can be used as a biomarker, there are studies that prove that it can serve that purpose. These questions can arise due to the type of screening method chosen or the combination of neurodegenerative diseases. Likewise, the results of Aβ quantification are very different. Some of the possible reasons are the barrier between blood and brain (BBB), the inability to measure the oligomeric form of the β-amyloid or the use of inappropriate antibodies in the protocols of ELISA [33] (Figure 4).

This systematic review includes 51 articles (Figure 2). Since the method of analysis is very important to identify the different biomarkers, we examined the different methods in all articles. For us, the Receiver Operating Characteristic (ROC) analysis was very important since it reports the sensibility and sensitivity values of the different methods. However, the articles which cited a ROC curve were 4% of all articles. Moreover, some articles promised better results in a specific method of analysis. Almost 15% of the articles talked about pre-treatment. 2% of the articles mentioned air plasma pre-treatment, the same applies to hexafluoroisopropanol and trifluoroacetic acid, gallic acid 2%, trypsin 2%, PBS buffer 2%, formic acid 4%, acid conditions 2%, and TSA 2% (Table 2, Figure 3 and Figure 4). Similarly, the ELISA analysis method was of particular importance to us and we found that almost 51% of the articles used ELISA to diagnose and measure the relevant biomarkers. These include: ELISA, single-analyte ELISA, sandwich ELISA, and two-site ELISA (Figure 4 and Figure 5, Table 2).

The biomarker dilemma is multidimensional and encompasses the need for a deep understanding of the aggregation/oligomerization process, their dynamic and their molecule networks (Figure 4). The challenge is to combine methodologies in order to explore the process of oligomerization and the co-aggregation network. The biophysical techniques such as electron microscopy, atomic force microscopy absorbance, fluorescence, Circular dichroism (CD), small-angle X-ray scattering and mass spectrometry have illustrated the structural features of the oligomeric species. For instance, we now know that the soluble amyloid oligomers—the most cytotoxic and the cause of death of target cells (in the brain, pancreas, etc.)—of conformational diseases are characterized by [11,15]:

(a) Low Molecular weight (trimers (10–15 kDa), hexamers (24–30 kDa) [20].
(b) Contain β-sheet-rich structures [6,11].
(c) Have similar immunogenic reactivities regardless of the target protein [11,15,87].
(d) Strong cytotoxicity [11,15].
(e) Can be formed by monomers (protein misfolding) or by fibril structures halting self-catalysis [1,88].
(f) The anti-amyloid oligomer antibody (A11) distinguishes them [11,15].
(g) Can be monitored by TEM or AFM [15]

This means that a way to overcome the biomarker dilemma while avoiding the polymorphism of oligomeric species is to concentrate on those oligomers with low molecular weight that are related to cellular apoptosis and may have a role to play in the developing stages of conformational diseases.

It is widely acknowledged that one of the most important factors to have a homogeneous population of soluble oligomers is to start with completely solvated stock solutions that lack fibril seeds using, for instance, trifluoroacetic acid NaOH or fluorinated alcohols. Low pH (pH 2.5–4) and low ionic strength is also generally useful. One general means of stabilizing the oligomers after they have been formed is to add NH_4_OH to a final concentration of 0.1%, (final pH 9.5–10.5). Brief sonication of the stock solutions for 30 s can serve to disperse and homogenize the samples [87].

In order for these conditions to be used with biological samples (serum, plasma, cerebrospinal fluid, etc.) there would have to be a way to universalise the pre-treatment, this would make it easier to get homogenous population of soluble oligomers. This pre-treatment would have to be cost-effective and easy to implement in diagnostic laboratories in hospitals.

The universalization of pre-treatments could potentially solve the ELISAs’ limitations as well as those of newer technologies such as electrochemical or optic biosensors.

Since PET studies were complimentary to the immunoassays analysis, the percentage of articles that cited the use of PET and SPECT were almost a 4% of the selected articles (Figure 3 and Table 2). Spectroscopic methods also played an important function in the research, since they are a new wave in the β-amyloid and Alzheimer’s disease intervention, 45% of the articles used for this study mentioned that they used a spectroscopic method. These include: Elecsys β-amyloid assay, Congo Red, fluorescent microscopy, thioflavin T, UV-visible spectroscopy, CD, AFM, FTIR, MS, TEM, SEC, Nile Red, MRI, single photon emission, computed tomography, and MMSE, being thioflavin T the most common one used (Table 2).

Even though ELISA had some preference in the study, some other articles cited using other immunoassays like western blot, innotest, innotest hTau-Ag, immunodot, homogeneous time-resolved fluorescence (HTRF), dot blot, XMAP, immunoprecipitations, multiples, immunoblot, and immunofluorescence microscopy. These accounted for almost 64% of all articles (Table 2). Lastly, genetic tests were used in other articles. These are almost the 4% of all articles used for this review. Some examples of these were PCR and PMCA.

Some of the clinical studies that have sought treatment have postulated a number of molecules capable of inhibiting β-amyloid protein. Small molecules like 4-aminophenol, resveratrol, myricetin, curcumin, caffeine, and immunization therapy AN1792 [33], are examples of possible treatment options. Gallic acid [57] and α-mangostin [38] have also been investigated for the same purpose. Other treatment alternatives are the chaperones which are expressed in situations of stress. This treatment has been shown to have positive effects on the pathogenesis of Alzheimer’s disease. Similar treatments like the use of the protein maltose binding protein (MBP) which is believed to have chaperone-like functions [63] (Table 2).

Other suggested treatment options include immunization. There are two types: Active and passive. Active immunization is much more cost-effective since a reduction of oligomers is evident, thanks to the development of more than 600 monoclonal anti-Aβ antibodies which destroy β-amyloid epitopes [32]. Some of them are A11, OC, Aapf, Anti-ADDL, Anti-ASPD, anti-globulomer and Gammabody [65]. These include the immunotherapeutic drug solanezumab [11], which identifies monomers and non-oligomers or fibres. However, this has a greater affinity to oligomers. This will have a great impact on achieving more precise diagnoses in ELISAs. Immunization with the Aβ42 oligomer conformation-sensitive serum antibodies, inhibits Aβ42 oligomer-mediated neurotoxicity in vitro, was effective in mitigate AD-like pathology and cognitive decline in 3× Tg-AD mice in vivo. This test has proven to be highly efficient in mice [38] (Table 2 and Figure 3).

In addition to the postulated treatments, it is important to mention that the investigation of molecules that prevent interaction and instability of the mitochondrial membrane by poorly folded species have considered the polyphenols with the help of curcumin, quercetin and resveratrol [72]. Another possible treatment is the administration of PTI-125. This already provided in triple-transgenic AD mice [38]. However, with this review it is important to mention that induced metal ions and A-BSA/A-HSA—as inhibitor of β-amyloid aggregation—are considered to be a potential, safe strategy for AD treatment. Yet, this method is still controversial [30] (Figure 3).

Recently our group demonstrated a family of pharmaco-chaperones that potentially treats the conformational diseases at various physio-pathological steps. For example, in chronic stage it is better to use chaperone A-D, which stabilizes the native and fibril structures halting self-catalysis. All the pharmaco-chaperones are able to protect and recondition the cerebellar granule cells from the apoptosis produced by the hIAPP20-29 fragment [22] (Figure 3).

The role of amyloid oligomers is multidimentional because they imply that they can be used for study, potentially be a therapeutic target, and relevant in the therapeutic drug monitoring in CDs.

ELISA has proved to be a reliable, accessible, non-invasive and cost-effective alternative for screening purposes as well as for post-treatment monitoring of several diseases. If we overcome the biomarker dilemma the same assay can be used in clinical trials to help assess the efficacy and adherence of therapy in CDs and give valuable feedback to healthcare personal and patients.

It is hoped that this review could open the door for the development of novel diagnostic methods and therapeutic agents, that will encourage research in reverse engineering and improve the control of the devastating global changes caused by conformational diseases.

## 4. Materials and Methods

A systematic review was carried for this study (Figure 1 and Figure 2) following a series of techniques described in the Participants/Intervention/Comparison/Outcome (PICO) [89] and Preferred Reporting Items for Systematic Reviews and Meta-Analyses (PRISMA) [90] protocols. These documents can be obtained as supplemental files. No ethical approval or letter of individual consent was required for this research.

### 4.1. Data Sources and Searches

The electronic databases used were PubMed, Embase and BVsalud. The search process started on June 2017 and came to an end in August 2017. According to the PICO methodology (Figure 1), the terms used to perform the search within these databases were:

- **P** (participants): Humans, animals, Alzheimer, Diabetes Mellitus, conformational diseases, amyloid oligomers, amyloid-β, oligomers, conformation.
- **I** (intervention): Immunoassays, Western Blot, Immunodot, ELISA, Enzyme-linked immunosorbent assay, plasma, serum, spreading, blood transmission.
- **C** (comparison): Method comparison and cost-effectiveness.
- (outcome): Diagnostic methods, diagnostic kit, diagnostic, treatment, therapy.

The concepts with similarity were searched with ‘OR’ and within the groups of each element of the PICO research, they were searched with the word ‘AND’. Next, a diagram was constructed in order to show the history of searches and concepts used (Figure 1). This figure describes in full detail the searching strategy in the PubMed database as well as all the key words used. Moreover, it includes the number of resulting articles. Subsequently, the results obtained from these searches were recorded in a Word document. The references themselves were then downloaded onto the Mendeley database.

### 4.2. Eligibility Criteria

In order to obtain the most relevant results, the selection process of the articles that would be taken into account was divided into three groups (Figure 2). The first round of selection consisted of discarding certain articles based on the type of document; for instance, if the documents were letters or conference notes they were discarded. The documents that predated 1989 were also discarded as well as those articles that were found to be irrelevant to the topic upon reading the abstract. The resulting references were individually downloaded in PDF format and stored in preparation for the second stage of the selection process.

### 4.3. Data Extraction and Quality Assessment

We evaluated all the papers from the first selection step, according to the following points: The articles to be taken into consideration had to have clear objectives and research questions, definitions of the concepts to be measured, valid and reliable measuring instruments, a detailed description of the method, information on the size and type of the target population, information on the number and characteristics of the subjects participating in the study, analysis of missing values, and adequate statistical analysis [37] (Figure 2). Each requirement was worth 12.5 points and so, each article was given a grade out of 100 points. To organize this data, a table with the most relevant information of each article and its score was made. Table 1 includes the author's name and year of publication, place of experiment, population size, population characteristics, experiment design and quality score.

Those articles with a score above 75 points were considered for deeper analysis and are listed in Table 2, which includes setting, target molecule, method of analysis, target population, clinical data, sensibility and specificity and pre-treatment.

## 5. Conclusions

Amyloid-β is a protein that still raises many questions in relation to the development of serious diseases like AD and diabetes mellitus. A series of experiments have developed techniques like ELISA, western blot and immunodot, yet these techniques lack uniformity and standardization. Moreover, the sample acquisition results are sometimes costly and invasive. Even though this is true, a series of studies examined in this review confirm the use of β-amyloid oligomers as a biomarker for the diagnosis of conformational diseases. Alternate techniques like imaging and histological tests have been used, however these are high-cost. For this reason, it is important to continue research in this area, even when treatment opportunities have already been achieved.

## Figures and Tables

**Table 1:** An overview of the included articles with the quality assessment.

First Author/Year of Publication	Setting	Target Population and Sample Size	Target Molecule	Method	Quality (%)
Andreasen, et al. 1999 [39]	Sweden	16 MCI-AD patients and 15 age-matched controls	CSF Aβ42 and CSF T-tau	Longitudinal MCI-control study/Elisa	100
Arai, et al. 1995 [41,42]	Japan	201 CSF samples, 87 patients with Alzheimer’s disease (AD) and 114 patients without neurological diseases	Microtubule-associated protein tau in CSF	Elisa	75
Benedett, et al. 2015 [43]	Canada	417 participants and 174 additional patients with samples of CSF and P-Tau	Aβ	SUVR and PET	87.5
Bittner, et al. 2015 [35]	Roche Diagnostics	372 Human CSF with diagnosed AD	Aβ	Elecsys β-amyloid assay (electro chemiluminescence immunoassay)	100
Blennow, et al. 2014 [27]	United States	Humans	Cerebrospinal fluid biomarkers such as B-amyloid, Tau and Tau phosphorylated for Alzheimer’s detection	Article review	75
Bouwman, et al. 2007 [44]	The Netherlands	59 MCI patients, 30 of them developed AD	CSF Aβ42 and CSF T-tau	Longitudinal MCI study	100
Breydo, et al. 2016 [24]	United States	Synthetic Aβ	Aβ40 oligomers FOs and PFOs	Raman, FTIR, CD spectroscopy and Western Blot	62.5
Brys, et al. 2007 [45]	United States	65 MCI patients, 22 of who later developed AD and 21 controls	CSF Aβ42, Aβ42/Aβ40 ratio, CSF T-tau, CSF P-tau231	Elisa and Innotest hTAU antigen kit	100
Bush, et al. 1992 [31]	Germany	Human platelets and plasma samples of patients diagnosed with AD	APP	Western Blot and Immunodot	87.5
Capule, et al. 2012 [36]	United States	Synthetic β-amyloid proteins and AD Aβ samples	Binding molecules to Aβ	Elisa	87.5
Chetri et al. 2015 [46]	India	Cultivated Aβ	Cultivated Aβ	Cloning of Aβ with PCR	62.5
Clarke, et al. 2000 [47]	United Kingdom	Antibodies and Aβ from humans and rodents	Aβ	HTRF immunoassay	87.5
Condello, et al. 2017 [48]	United States	Humans samples diagnosed with AD	Aβ and Tau aggregates	Therapy and diagnostic options revision for Alzheimer’s	87.5
Despa, et al. 2012 [25]	United States	Left ventricular homogenates of obese, non-obese and diabetic patients and rodents	Amylin	Western Blot/Dot Blot	100
Doran, et al. 2015 [49]	United States	Antigen substitutes	Antigens capable of identifying sites of antibodies. Types: OBOC of animals or humans control and with disease of interest.	ELISA measurement	75
Giacomelli, et al. 2017 [33]	Italy	Human post-mortem brain, plasma, platelets, CSF, RBC, samples and AD mice	Aβ,tau and a-syn	Bibliographic analysis	75
Gustafson, et al. 2007 [50]	Sweden	55 cognitively healthy women	CSF Aβ42	Longitudinal cohort study and Sandwich ELISA (Innotest hTAU-Ag)	100
Hansson, et al. 2006 [51]	Sweden	137 MCI patients, 57 of whom developed AD	CSF Aβ42, high CSF T-tau, and CSF P-tau181	Luminex xMAP technology	100
Hansson, et al. 2007 [52]	Sweden	137 MCI patients, 57 of whom developed AD	Aβ42/Aβ40 ratio	Elisa	100
Hampel, et al. 2004 [53]	Germany	52 MCI patients, 93 AD patients and 10 controls	CSF Aβ42 and CSF T-tau	Elisa	100
Herukka, et al. 2005 [54]	Finland	78 MCI patients, 23 of whom developed AD, 46 controls	CSF Aβ42, CSF T-tau, and CSF P-tau181	Elisa	100
Herukka, et al. 2007 [55]	Finland	79 MCI patients, 33 of whom developed AD, 60 controls	CSF Aβ42, CSF T-tau, and CSF P-tau181	PCR and Elisa	100
Iwatsubo. 1998 [28]	Japan	6 patients with Beta APP 717, 44 patients with sporadic AD, 22 controls of neurological diseases and 15 controls without neurological disease	C-terminus of β-amyloid 42 and β-amyloid 40	Two-site Elisa	87.5
Janssen, et al. 2015 [56]	Belgium	Aggregated β-amyloid oligomers from mouse brain and human CSF	β-amyloid	Samples exposed to a pretreatment with TFA, FA and HFIP with ELISA/Western Blot (SDS-PAGE)	75
Jayamani, et al. 2014 [57]	India	Synthetic insulin and Gallic acid	Insulin in vitro and Aβ fibril formation	UV-Visible spectroscopy, ThT fluorescence spectroscopy, CD, Fourier-transformed infrared (FTIR) spectroscopy, and fibril morphology using atomic force microscopy (AFM)	87.5
Kepp, et al. 2017 [29]	Denmark	Patients with Alzheimer’s disease	Metal ions and β-amyloid	Bibliographic review	75
Khan, et al. 2017 [58]	United States	Human Aβ40 synthetic peptides	Aβ40	Dichroism and Fluorescence emission spectroscopy	62.5
Kim, et al. 2014 [59]	Korea	Human plasma	Synthetic Aβ40	SRM-MS and antibody-free spectrometry	87.5
Klaver, et al. 2011 [60]	United States	328 Serum antibodies with AD, MCI or ICU patients	β-amyloid and antibodies	Western Blot and Elisa	87.5
Kuo, et al. 2017 [61]	Taiwan	Hen lysozyme	Amyloid fibrils and erythrosine B	Molecular docking and molecular dynamics simulations	62.5
Li, et al. 2007 [62]	United States	43 controls, 4 of whom developed MCI	T-tau/Aβ42 ratio	Luminex reagents	100
Mangione, et al. 2016 [63]	Italy	Chaperone Hsp60	Aβ40	CD, TEM, AFM and SEC	75
Mattsson, et al. 2009 [40,64]	Sweden	750 MCI patients, 271 of whom developed AD	CSF Aβ42, CSF T-tau, and CSF P-tau181	Sandwich Elisa	100
Murakami. 2014 [65]	Japan	Alzheimer’s disease patients	Aβ oligomers	Immunotherapy	87.5
Niedowicz, et al. 2012 [66]	United States	23 controls age 87 years average, 22 patients with Alzheimer's average age 85	Aβ	Elisa	75
Ordóñez-Gutiérrez, et al. 2016 [32]	Spain	ImmunoPEGliposomes with β-amyloid in brain cells and in vitro phagocytes in mice aged 16 months and 10 months of age	β-amyloid peptides	Elisa and Western Blot	87.5
Permanne, et al. 1995 [67]	France	17 elderly with AD and no AD	Aβ	Dot-blot and Western Blot	100
Plagg, et al. 2015 [68]	Austria	Platelets of mice with hypercholesterolemia and 43 humans with and 30 controls	APP	Elisa and Western Blot	87.5
Read, et al. 1992 [34]	The Netherlands	4 CSF samples from patients with dementia	APP	Elisa	75
Riemenschneider, et al. 2002 [69]	Germany	4 ventricular and lumbar CSF samples ventricular with AD	APP	Elisa	100
Ringman, et al. 2008 [70]	United States	CSF biomarker results were compared in 7 asymptomatic carriers of familial AD (FAD)-associated mutations and four non-carriers	CSF Aβ42, Aβ42/Aβ40 ratio, CSF T-tau, and CSF P-tau181	Elisa	100
Rogeberg, et al. 2015 [71]	Norway	19 of the Alzheimer’s CSF samples and 19 CSF healthy controls	Aβ	Mass spectrometry (MS), sandwich Elisa, Immunoprecipitation	87.5
Roqanian, et al. 2017 [72]	Iran	Cerebral rat samples	HEWL Oligomers	ThT fluorescent assay, and Nile red binding assay	100
Ruiz, et al. 2015 [73]	Mexico	Aggregated β-amyloid fibrils	Aβ fibrils	Surface tension and DLS	75
Salvadores, et al. 2014 [74]	United States	50 samples of CSF patients with AD + 30 healthy control patients and 39 other neurological diseases	Aβ oligomers/PrP	PMCA	87.5
Sharoar, et al. 2013 [75]	Bangladesh	Samples of human neuroblastoma cells in vitro	Aβ	Th-T assay, CD spectroscopy, Immunoblot and Dot blot	75
Shaw, et al. 2009 [76]	United States	196 MCI patients, 37 of whom developed AD	CSF Aβ42, CSF T-tau, and CSF P-tau181	Multiplex immunoassay; xMAP Luminex	100
Sengupta, et al. 2016 [11]	United States	Alzheimer’s disease	Aβ	Bibliographic Review	87.5
Skoog, et al. 2003 [77]	Sweden	57 cognitively normal controls underwent LP and were followed for 3 years	CSF Aβ42	Sandwich Elisa	100
Spiegel, et al. 1992 [78]	United States	22 blood samples from control patients aged 50 years on average and 59 patients with chronic hemodialysis	Aβ	'Rocket' immunodiffusion test	100
Stern, et al. 1990 [79]	United States	Blood samples and blood antibodies	Aβ	Western Blot	75
Stomrud, et al. 2007 [80]	Sweden	57 cognitively normal controls underwent LP and were followed for 3 years	CSF Aβ42	xMAP technology and the INNO-BIA AlzBio3 kit	100
Tamaoka, et al. 1997 [81]	Japan	CSF of AD patient samples and 34 without AD	Aβ	Elisa and sandwich Elisa	100
Wang, et al. 2012 [82]	China	Primary rat cerebral cortical neurons	β-amyloid with alfa-M	Dot blot assay, Western Blot and ThT fluorescence	75
Wang, et al. 2016 [38]	China	Brain samples of passively immunized 3× Tg-AD mice and rabbits control and wild-type	Dynamin 1	Western Blot, Immunoblots, Dot Blot and Elisa	75
Wang, et al. 2017 [83]	United States	Triple-transgenic (3× Tg) AD mice and humans samples diagnosed with AD	Solubilized immunoprecipitates	Western Blot	87.5
Xie, et al. 2017 [30]	China	Human neuroblastoma SH-SY5Y cells	Aβ aggregates	Thioflavin T fluorescent assay	87.5
Yang, et al. 2013 [84]	United States	90 samples of human brain and CSF samples with AD and no AD and transgenic mouse brains	Aβ oligomers	Sandwich Elisa, o-Elisa, Immunoprecipitation and Western Blot	87.5
Yang, et al. 2014 [85]	United States	26 APPswe/PS1 transgenic mice blood samples	Gelsolin	Western Blot and sandwich Elisa	75
Zetterberg, et al. 2003 [86]	Sweden	53 MCI patients, 22 of whom developed AD	CSF Aβ42, CSF T-tau, and CSF P-tau181	Electroencephalogram, magnetic resonance imaging, single photon emission, computed tomography and MMSE	100
Zhao, et al. 2009 [26]	China	8 nondiabetic control subjects, 8 type 2 diabetic cases without islet amyloid, and 8 type 2 diabetic patients with islet amyloid	Aβ oligomers	Immunofluorescent microscopy and autopsy	100

**Table 2:** An overview of the included articles with the study characteristics.

Reference	Setting	Target Molecule	Method of Analysis	Target Population	Clinical Data	Sensibility and Specificity	Pretreatment	Antibodies
Andreasen, et al. 1999 [39]	Sweden	CSF Aβ42 and CSF T-tau	Elisa	16 MCI-AD patients, 15 age-matched controls	Low CSF Aβ42, high CSF T-tau associated with AD	Sensibility: 88%	IA	-Antibodies: 21F12 and 3D6 -Kit: Innotest β-amyloid (1–40) Innogenetics
Arai, et al. 1996 [41,42]	Japan	Microtubule-associated protein tau in CSF	Sandwich Elisa and Western Blot	114 patients non-AD neurological diseases, 22 normal subjects	CSF tau increased in AD patients compared with non-AD neurological disease	Sensitivity and specificity missing	IA	-Not mentioned
Bittner, et al. 2015 [35]	Roche Diagnostics	Aβ	Elecsys β-amyloid assay	372 Human CSF	Elecsys β-amyloid 42 has high analytical performance that improves biomarker-based AD diagnosis	Sensitivity and specificity missing	IA	
Bouwman, et al. 2007 [44]	The Netherlands	CSF Aβ42 and CSF T-tau	Innotest β-amyloid1-42 and Innotest hTau-Ag	59 MCI patients, 30 of whom developed AD	Patients with abnormal values at baseline had higher risk of developing AD.	Sensitivity and specificity missing.	Pretreatment missing.	
Brys, et al. 2007 [45]	United States	CSF Aβ42, Aβ42/Aβ40 ratio, CSF T-tau, CSF P-tau231	Innotest hTAU antigen kit; sandwich Elisa for P-tau231	65 MCI patients, 22 of whom developed AD, 21 controls	All biomarkers were statistically significant predictors of the decline from MCI to AD with P-tau231 and T-tau the strongest univariate predictors.	Sensitivity 68–86%, specificity 60–91%	Pretreatment missing.	-Antibody: Monoclonal antibody 6E10 -Kit: INNOTEST hTAU antigen kit (Innogenetics®, Gent, Belgium).
Bush, et al. 1992 [31]	Germany	APP	Western Blot; Immunodot	Human platelets, human plasma of AD cases	β-amyloid deposition may result in failure of APP	Sensitivity and specificity missing	Pretreatment missing	
Capule, et al. 2012 [36]	United States	Binding molecules to Aβ	Elisa	96 ELISA plates of Synthetic beta amyloid proteins and AD Aβ samples	Protocol overcomes many limitations of previously reported spectroscopic or radioactivity assays and facilitate the screening and evaluation of a more structurally diverse set of amyloid-targeting agents	Sensitivity and specificity missing.	Air plasma	-Antibody: 6E10 -Kit: not mentioned
Clarke, et al. 2000 [47]	United Kingdom	Aβ	Homogeneous time-resolved fluorescence (HTRF) immunoassay	Synthetic β-amyloid proteins and antibodies from humans and rodents	This assay allows specific, direct quantitation of Aβ peptides in cell culture medium, plasma, cerebrospinal fluid and brain tissue extracts.	Sensitivity and specificity missing	Pretreatment missing	
Despa, et al. 2012 [25]	United States	Amylin	Western Blot/Dot Blot	Left ventricular homogenates of humans and rodents with DM2 and controls	Hyperamylinemia promotes amylin deposition in the heart, causing alterations of cardiac myocyte structure and function.	Sensitivity and specificity missing.	Pretreatment missing.	
Giacomelli, et al. 2017 [33]	Italy	Aβ,tau and a-syn	Congo red Fluorescent microscopy, Thioflavin-T, Elisa, SPECT, PET and Western Blot	Human post-mortem brain, plasma, platelets, CSF, RBC, samples and AD mice	Biomarkers establishment and assessment is important for diagnosis and therapeutic options	IA	IA	
Gustafson, et al. 2007 [50]	Sweden	CSF Aβ42	Sandwich ELISA and Innotest hTAU-Ag	55 cognitively healthy women	Low levels of CSF Aβ42 predicted cognitive decline.	Sensitivity and specificity missing.	Pretreatment missing.	-Antibody: not mentioned -Kit: Innotest β-amyloid 1-42; Innogenetics, Zwijndrecht, Belgium
Hansson, et al. 2006 [51]	Sweden	CSF Aβ42, high CSF T-tau, and CSF P-tau181	Luminex xMAP technology	137 MCI patients, 57 of whom developed AD	Concentrations of T-tau, P-tau181, and Aβ42 in CSF are strongly associated with future development of Alzheimer’s disease in patients with MCI.	Sensitivity 95%, specificity 83%, PPV 81%, NPV 96%	IA	
Hansson, et al. 2007 [52]	Sweden	Aβ42/Aβ40 ratio	Elisa	137 MCI patients, 57 of whom developed AD	Amyloid precursor protein metabolism is disturbed in early sporadic AD and points to the usefulness of the Aβ42/Aβ40 ratio as a predictive biomarker for AD.	Sensitivity 87%, specificity 78%	Pretreatment missing.	-Antibodies: W02 (epitope AB 5–8), detection anti-bodies G2–10 for Aβ40 and G2–13 for Aβ42 -Kit: The Genetics Company
Hampel, et al. 2004 [53]	Germany	CSF Aβ42 and CSF T-tau	Elisa	52 MCI patients, 93 AD patients and 10 controls	CSF tau and Aβ1-42 may be useful biomarkers in the early identification of AD in MCI subjects.	Sensitivity 59–83%, specificity 90–100%	Pretreatment missing.	-Antibody: not mentioned -Kit: Innotest β-amyloid 1-42 and Innotest hTAU-Ag, Innogenetics, Zwjindrecht, Belgium
Herukka, et al. 2005 [54]	Finland	CSF Aβ42, CSF T-tau, and CSF P-tau181	Elisa	78 MCI patients, 23 of whom developed AD, 46 controls	The most predictive assay for AD among the patients with MCI was the combination of Aβ42 and P-tau.	Sensitivity 91%, specificity 56%	Pretreatment missing.	-Antibody: not mentioned -Kit: Innogenetics, Ghent, Bel-gium
Herukka, et al. 2007 [55]	Finland	CSF Aβ42, CSF T-tau, and CSF P-tau181	PCR and Elisa	79 MCI patients, 33 of whom developed AD, 60 controls	Low levels of CSF Aβ42 predicted progression to AD.	Sensitivity and specificity missing.	Pretreatment missing.	-Antibody: not mentioned -Kit: Innogenetics, Ghent, Belgium
Iwatsubo, 1998 [28]	Japan	C-terminus of Aβ 42 and β-amyloid 40	Two-site Elisa	6 patients with Beta APP 717, 44 patients with sporadic AD, 22 controls of neurological diseases and 15 controls without neurological disease	Levels of Aβ, especially those of Aβ42 are altered in the plasma of patients with AD, including carriers of APP717 mutation that is linked to familial AD.	Sensitivity and specificity missing.	Pretreatment missing.	
Janssen, et al. 2015 [56]	Belgium	β-amyloid	Elisa and Western Blot	Aggregated β-amyloid oligomers from mouse brain and human CSF	Chemically pre-treating samples to disaggregate oligomers can (partially) recover the signal loss.	7.81 and 500 pg/mL	Trifluoroacetic acid, formic acid or HFIP	-Antibody: 6E10 -Kit: Human Amyloid β (1-x) Assay kit (IBL International)
Jayamani, et al. 2014 [57]	India	Insulin in vitro	UV-Visible spectroscopy, ThT fluorescence spectroscopy, CD, Fourier-transformed infrared (FTIR) spectroscopy, and fibril morphology using atomic force microscopy (AFM)	Synthetic insulin and Gallic acid	Gallic acid can inhibit insulin Aβ fibril formation in vitro	Sensitivity and specificity missing.	Gallic acid	
Kepp, et al. 2017 [29]	Denmark	Metal ions and β-amyloid	IA	Patients with Alzheimer’s disease	The metal-Aβ interactions have elements of both gain of toxic function. Possible treatments for β-Amyloid accumulation: metal chelation, treatment with anti-oxidant and anti-inflammatory molecules.	IA	IA	
Kim, et al. 2014 [59]	Korea	β-amyloid	Mass spectrometry (MS)-based quantification	Human plasma	β-amyloid can be measured regardless of the conformational status of the biomarker	Sensitivity and specificity missing.	Trypsin	
Klaver, et al. 2011 [60]	United States	β-amyloid and antibodies	Western Blot and Elisa	328 Serum antibodies with AD patients, subjects with mild cognitive impairment, and aged non-cognitively impaired individuals	Hypothesis that reduced levels of anti- Aβ antibodies might contribute to AD’s pathogenesis not proven.	Sensitivity and specificity missing.	Pretreatment missing	-Antibody: 6E10 anti-Aβ antibody -Kit: not mentioned
Li, et al. 2007 [62]	United States	T-tau/Aβ42 ratio	Luminex reagents	43 controls, 4 of whom developed MCI	Individuals with high ratio had higher APOE ε4 allele frequency and higher risk of progression to MCI	High sensitivity and specificity	IA	
Mangione, et al. 2016 [63]	Italy	Aβ40	CD, TEM, AFM and SEC	Chaperone Hsp60	Hsp60 inhibits Aβ	Sensitivity and specificity missing	IA	
Mattsson, et al. 2009 [40,64]	Sweden	CSF Aβ42, CSF T-tau, and CSF P-tau181	Sandwich Elisa	750 MCI patients, 271 of whom developed AD	CSFA 42, T-tau, and P-tauidentify incipient AD with good accuracy	Sensitivity 83%, specificity 88% for MCI-AD versus controls; sensitivity83%, specificity 72% for MCI-AD versus all MCI cases	Pretreatment missing	-Innotest Phospho-Tau[181P] -Innotest-amyloid (1-42)
Murakami. 2014 [65]	Japan	Aβ oligomers	Immunotherapy	Alzheimer’s disease patients	Immunotherapy using anti-Aβ anti- body is a possible approach for AD treatment	IA	IA	
Niedowicz, et al. 2012 [66]	United States	Aβ	Elisa	Samples of patients with AD (22) and controls (23) and four different brain regions	Postmortem PiB binding is useful in distinguishing AD from control cases, SDS-soluble Ab measured by standard immunoassay was better.	Sensitivity of 100% and a specificity of 95.7%	PBS buffer	-Antibody: Ab9 (human sequence Ab1–16), Ab13.1.1, 12F4 (Covance, Princeton, NJ) y 4G8 -Kit: not mentioned
Ordóñez-Gutiérrez, et al. 2016 [32]	Spain	Aβ	Elisa and Western Blot	ImmunoPEGliposomes with β-amyloid in brain cells and in vitro phagocytes in mice aged 16 months and 10 months of age	Treatment lowered the ratio of phosphorylated Tau to total Tau. Therapeutic efficacy of immunoliposome treatment was superior to free monoclonal antibody administration.	Sensitivity and specificity missing.	Pretreatment missing	-Antibody: 6E10 anti-Aβ -Kit: Qubits Protein Assay Kit.
Permanne, et al. 1995 [67]	France	Aβ	Dot-blot and Western Blot	17 elderly with AD and no AD	Method detects amyloid-associated components such as apolipoprotein E.	High sensitivity	Formic acid	
Plagg, et al. 2015 [68]	Austria	APP	Elisa and Western Blot	73 patients with AD (43) and controls (30)	Decreased platelet APP isoforms in AD patients, APP beta altered in humans and mice with AD, lower EGF levels in human AD patients.	Highly sensitive.	Pretreatment missing	-Antibody: biotinylated antibody CD62P -Kit: sAPPb-w (highly sensitive) Assay Kit—IBL
Read, et al. 1992 [34]	The Netherlands	APP	Elisa	4 ventricular and lumbar CSF samples ventricular with AD	Low APP levels as a diagnostic marker for AD	Sensitivity and specificity missing.	Pretreatment missing	-Antibody: not mentioned -Kit: not mentioned
Riemenschneider, et al. 2002 [69]	Germany	CSF Aβ42 and CSF T-tau	Elisa	28 MCI patients, 10 of whom developed AD	Results indicate that altered tau and Aβ42 concentration can be detected in MCI patients but with pathological changes of AD	Sensitivity 90%, specificity 90%	Pretreatment missing	-Antibody: not metioned -Kit: Innogenetics, Zwjindrecht, Belgium
Ringman, et al. 2008 [70]	United States	CSF Aβ42, Aβ42/Aβ40 ratio, CSF T-tau, and CSF P-tau181	Elisa	CSF biomarker results were compared in 7 asymptomatic carriers of familial AD (FAD)-associated mutations and four non-carriers	Asymptomatic FAD mutation carriers had abnormal CSF biomarkers already in their 30 s	Sensitivity and specificity missing	Pretreatment missing	-Antibody: Takeda BAN50/BA27 and BNT77/BC05 antibodies -Kit: not mentioned
Rogeberg, et al. 2015 [71]	Norway	Aβ	Mass spectrometry (MS), sandwich Elisa, Immunoprecipitation	19 samples of CSF of AD patients and 9 control healthy patients	Method could be used to assess disease-modifying therapies directed at Aβ production or degradation.	Sensitivity and specificity missing	Pretreatment missing	-Antibodies: 4G8, 6E10, and a 12EF325 mid-domain antibody -Kit: Innotest hTau Ag, Phospho-Tau 181P, β-amyloid (1-42)
Roqanian, et al. 2017 [72]	Iran	HEWL Oligomers	ThT fluorescent assay, Nile red binding assay	Cerebral rat samples	Polyphenols frequently interacting with amyloid aggregates may serve as a therapeutic approach for amyloid-related diseases.	Sensitivity and specificity missing.	IA	
Ruiz, et al. 2015 [73]	Mexico	Aβ fibrils	DLS	Aggregated β-amyloid fibrils	IA	Sensitivity and specificity missing.	IA	
Salvadores, et al. 2014 [74]	Italy	Aβ oligomers/PrP	PMCA	50 samples of CSF patients with AD + 30 healthy control patients + 39 other neurological diseases	AD patients from control individuals affected by a variety of other neurodegenerative disorders or nondegenerative neurological diseases	Sensitivity of 90% and specificity of 92%	IA	
Shaw, et al. 2009 [76]	United States	CSF Aβ42, CSF T-tau, and CSF P-tau181	Multiplex immunoassay and xMAP Luminex	196 MCI patients, 37 of whom developed AD	CSF Aβ1-42 was the most sensitive biomarker for AD in the autopsy cohort of CSF	CSF T-tau/Aβ42 had a sensitivity of 89% for MCI cases with progression to AD	Pretreatment missing.	
Skoog, et al. 2003 [77]	Sweden	CSF Aβ42	Sandwich Elisa	35 non-demented 85 year olds underwent LP and were followed for 3 years	Low levels of CSF Aβ42 predicted progression to dementia	Sensitivity and specificity missing.	Pretreatment missing.	-Antibody: 3D6 -Kit: not mentioned
Stomrud, et al. 2007 [80]	Sweden	CSF Aβ42	xMAP technology	57 cognitively normal controls underwent LP and were followed for 3 years	Low levels of CSF Aβ42 predicted cognitive decline	Sensitivity of 71.4% and a specificity of 75.7%	IA	
Tamaoka, et al. 1997 [81]	Japan	Aβ	Elisa and sandwich Elisa	CSF of AD patient samples and 34 without AD	CSF-Aβ42(43) could reflect increased amino terminal truncations and/or modifications of Aβ42(43) in AD brains	Sensitivity and specificity missing.	Pretreatment missing.	-Antibodies: BNT77 (anti-Aβ11-28) and BAN50 (anti-Aβ1-16) -Kit: not mentioned
Wang, et al. 2012 [82]	China	β-amyloid with alfa-M	Dot blot assay, Western Blot and ThT fluorescence	Primary rat cerebral cortical neurons	a-M could be a great potential candidate for AD treatment	Sensitivity and specificity missing.	Pretreatment missing.	
Wang, et al. 2016 [38]	China	Dynamin 1	Western Blot, Immunoblot, Dot Blot and Elisa	Brain samples of passively immunized 3× Tg-AD mice control and wild-type	Passive immunization with Aβ42 possible treatment	Sensitivity and specificity missing.	Pretreatment missing.	-Antibody: not mentioned -Kit: Biosource ELISA kits (Invitrogen, Carlsbad, CA, USA)
Wang, et al. 2017 [83]	United States	Solubilized immunoprecipitates	Western Blot	Triple-transgenic (3× Tg) AD mice	PTI-125 is the first therapeutic candidate for AD	Sensitivity and specificity missing.	Pretreatment missing.	
Xie, et al. 2017 [30]	China	Aβ aggregates	Thioflavin T fluorescent assay	Human neuroblastoma SH-SY5Y cells	A-HSA worked as a bifunctional inhibitor against Cu^2+^-mediated Aβ42 aggregation and cytotoxicity under a mildly acidic condition	Sensitivity and specificity missing.	Acid conditions	
Yang, et al. 2013 [84]	United States	Aβ oligomers	Sandwich Elisa, Immunoprecipitation and Western Blot	Human brain samples with AD and no AD and transgenic mouse brains	New o-ELISAs method for biomarker AD	Sensitivity and specificity missing.	IA	-Antibodies: (MAb) 266 to the Aßmidregion (residues 13–28) or MAb, 3D6 (to residues 1–5), Mab 4G8 (to residues 18–22; D6 or MAbNAB61, MAbs266 and 3D6 were kindly provided by Elan, plc -Kit: not mentioned
Yang, et al. 2014 [85]	United States	Gelsolin	Western Blot and sandwich Elisa	26 APPswe/PS1 transgenic mice blood samples	Gelsolin decreases Aβ	Sensitivity and specificity missing.	TSA	-Antibody: 6E10 -Kit: not mentioned
Zetterberg, et al. 2003 [86]	Sweden	CSF Aβ42, CSF T-tau, and CSF P-tau181	Electroencephalogram, MRI, single photon emission, computed tomography and MMSE	53 MCI patients, 22 of whom developed AD	Missing information.	Sensitivity 68%, specificity 97%, PPV 94%, NPV 81%	IA	
Zhao, et al. 2009 [26]	China	Aβ oligomers	Immunofluorescent microscopy	8 nondiabetic control subjects, 8 type 2 diabetic cases without islet amyloid, and 8 type 2 diabetic patients with islet amyloid	Large oligomers were spatially localized adjacent to amyloid fibrils and were associated with apoptosis	Sensitivity and specificity missing.	IA	
